# When to take the primary certification examination: sooner or later?

**DOI:** 10.1016/j.acpath.2024.100116

**Published:** 2024-03-22

**Authors:** Gary W. Procop, Tyler J. Sandersfeld, Mario Levesque, Ty McCarthy, Bonnie Woodworth, Steven H. Swerdlow

**Affiliations:** American Board of Pathology, Tampa, FL, USA

**Keywords:** Board, Certification, Examination, Timing

## Abstract

Most Pathology residents take the Anatomic Pathology and/or Clinical Pathology primary pathology certification examination(s) near the end of their final year of training (i.e., Spring), whereas some postpone the examination(s) to the Fall administration of that year or even later. We compared the Spring and Fall administration pass rates of initial primary certification candidates for those who graduated in the same year they took the examination. We also compared the pass rates of same-year graduates with individuals who postponed the examination for a year or more. We also surveyed the candidates regarding the reasons they chose the Spring or Fall administration. Candidates who chose the earlier (i.e., Spring) administration were more likely to pass compared with those who took the later Fall administration (p = 0.0026 for Anatomic Pathology; p = 0.0004 for Clinical Pathology). Delaying the certifying exams beyond the calendar year of residency graduation was associated with a higher failure rate (p < 0.0001 for both Anatomic and Clinical Pathology). The survey results suggest that residents often take their certification examinations earlier to not interfere with fellowship training, because it coincides with the completion of residency training, or it is expected by their program. Pathology residents are more likely to pass the primary certification examinations when they are taken closer to the end of training, rather than postponing it to a later administration. Pathology residency program directors should encourage residents, who are deemed ready, to take their certification examinations at the earliest possible administration.

## Introduction

Residents who complete Pathology training in an Accreditation Council on Graduate Medical Education (ACGME) accredited training program and have completed the requirements for primary certification by the American Board of Pathology most often attempt the Anatomic Pathology (AP) certification examination and the Clinical Pathology (CP) certification examination for a combined anatomic and clinical pathology (AP/CP) primary certification. There are also primary certifications in AP only and CP only. There are two examination administrations each year for primary certification. The earliest administration is in the Spring of the year that corresponds with the final months of residency training, whereas the second administration is in the Fall of that year, after the completion of residency training. Historically, most candidates choose to take the primary certification at the first (i.e., Spring) administration of the year in which they graduate.

Although the primary certification examinations given in the Spring administration is psychometrically equivalent to the exam given in the Fall of the same year, a tendency for a higher pass rate for the Spring candidates has been noted. We, therefore, undertook a comparison of the pass rates of recent graduates who took the AP and/or CP certifying examination(s) for the first time in the Spring compared with those who chose the Fall administration of the same year. We also assessed pass rates of those who postponed the exam(s) a year or more and surveyed individuals from the 2021 and 2022 examination administration regarding why they chose either the Spring or Fall administration.

## Materials and methods

### Quantitative analysis

The first portion of this study was limited to AP certification examination candidates and CP certification examination candidates, who attempted certification for the first time in the same year in which they finished residency training. This study was a retrospective review from 2015 to 2023, with the exclusions of 2020 because only a single primary certification examination administration was given that year due to the COVID-19 pandemic and 2022 because one form was administered to all candidates in the Spring and a different form was administered to all candidates in the Fall, making comparison between Spring and Fall candidates unreliable. The differences in pass rates between Spring administration candidates and Fall administration candidates were compared.

Similarly, we also studied the differences between AP certification examination candidates and CP certification examination candidates who took the exam at either administration of the year in which they finished residency training versus those who chose to postpone the exam a year or more.

The AP certification examination and CP certification examination are given and scored separately, so these were assessed independently. A chi-square test of independence was conducted to determine if there was a statistically significant difference in the pass rates of candidates who took their initial AP and/or CP certification examination(s) in the Spring (i.e., first administration) versus those who took the examinations in the Fall (i.e., second administration) of the same year in which they completed residency. The same approach was taken to assess if there were differences of statistical significance between candidates who took their initial AP and/or CP certification examination(s) the year in which they completed residency versus those who postponed the certifying examination(s) for a year or more.

The chi-square requires at least 10 expected observations in each group (i.e., the pass and fail categories). There were less than ten candidates who failed in the Fall primary certification administrations for many of the years studied, because far fewer candidates chose the Fall administration. Therefore, the pass/failure counts of candidates were compiled and compared from all years between 2015 and 2023 (excluding 2020 and 2022) for statistical analysis (Table 1, Table 2). P-values were considered significant when less than 0.05.

**Table 1:** Chi-square test of independence between same-year spring and same-year fall candidates for AP and CP exams.

	N	Pass	Fail	*x*^2^	*p*	Cohen's *d*
Spring AP	3540	3235 (91.38%)	305 (8.62%)	9.09	0.0026	0.362
Fall AP	169	143 (84.62%)	26 (15.38%)			
Spring CP	3375	3201 (94.84%)	174 (5.16%)	12.74	0.0004	0.489
Fall CP	163	144 (88.35%)	19 (11.65%)			

Notes: *x*^*2*^ = Chi-square statistic; *p* = probability of observed difference or greater if true difference is zero; Cohen's *d* = standardized effect size.

**Table 2:** Chi-square test of independence between same-year and nnext-year or greater candidates for AP and CP exams.

	N	Pass	Fail	*x*^2^	*p*	Cohen's *d*
Same-Year AP	3709	3378 (91.08%)	331 (8.92%)	115.03	<0.0001	0.740
Later-Year AP	352	256 (72.73%)	96 (27.27%)			
Same-Year CP	3538	3345 (94.54%)	193 (5.46%)	106.80	<0.0001	0.904
Later-Year CP	227	175 (77.09%)	52 (22.91%)			

Notes: *x*^*2*^ = Chi-square statistic; *p* = probability of observed difference or greater if true difference is zero; Cohen's *d* = standardized effect size.

Cohen's *d* effect sizes were calculated for all the comparisons with the standard interpretive effect ranges applied. Cohen's *d* effect sizes of <0.20 were considered negligible, 0.20–0.49 small, 0.50–0.79 medium, and 0.80 and above large.

### Qualitative analysis

Anecdotal conversations with residents have suggested that some candidates who choose the earlier administration may do so because the material is “fresh in their minds,” or so that studying for the examination does not interfere with fellowship training. Individuals who choose to postpone their primary certification examination(s) may do so to have more time to study. Therefore, we surveyed candidates from 2021 to 2022 to inquire why individuals chose the Spring or the Fall administration (Table 3). The responses to these surveys were coded and the major themes were determined by a manual response review, which correlated well with collation and categorization by a generative artificial intelligence tool (i.e. ChatGPT-3) (OpenAI, https://chat.openai.com/) (Table 4).

**Table 3:** Response rates for administration choice survey by time of initial examination.

	N Surveyed	N Responses	Response Rate
Spring 2021	450	120	26.7%
Fall 2021	8	3	37.5%
Spring 2022	433	134	31.0%
Fall 2022	10	3	30.0%

**Table 4:** Thematic analyses of survey responses.

Thematic Analyses		
Category Number	Theme	Percentage
Manual Thematic Analysis		
1	Complete while in residency when the information was fresh and to not distract from fellowship training/job	57.9%
2	Coincident with end of training and felt ready	11.9%
3	Convenience and to complete certification as soon as possible	8.7%
4	Program expectation	7.1%
5	Other/No response	14.3%

Generative AI Thematic Analysis (Limited to Four Categories)		

1	Timing with graduation and fellowship start	51.6%
2	Preparation and study time	24.2%
3	Avoiding interference with fellowship	16.2%
4	Program recommendation	8.0%

Generative AI Thematic Analysis (No Categorical Limitations)		

1	Alignment with fellowship or residency schedule	36.4%
2	Preparation and study time	19.8%
3	Avoiding distraction during fellowship	17.3%
4	Completion before starting a new position	7.5%
5	Freshness of knowledge	5.5%
6	Standard or recommended timing	5.1%
7	Desire to finish quickly	3.8%
8	Opportunity for a second attempt	2.2%
9	Cost considerations	1.9%
10	Specialized training	1.1%
11	Miscellaneous	4.4%

## Results

The compiled pass/fail rates for the AP certification examination candidates between 2015 and 2023, excluding 2020 and 2022, demonstrated that 91.4% (3235/3540) of Spring candidates passed the examination, whereas only 84.6% (143/169 of Fall candidates passed the examination (*x*^2^ = 9.09; *p* = 0.0026; Cohen's d = 0.362). The compiled pass/fail rates for the CP certification examination candidates between 2015 and 2023, excluding 2020 and 2022, demonstrated that 94.8% (3201/3375) of the Spring candidates passed the examination, whereas only 88.3% (144/163) of the Fall initial candidates passed the examination (*x*^2^ = 12.74; *p* = 0.0004; Cohen's d = 0.489). The effect sizes for each exam showed a small advantage toward candidates who took their first exam in the Spring (Table 1).

Individuals who took the initial AP certification examination and or CP certification examination in the year in which they completed training were far more likely to pass compared with those who postponed the examination(s) a year or more. The pass rate of same-year candidates on the AP certification examination was 91.1% (3378/3709), whereas the pass rate of candidates who postponed the examination a year or more was 72.7% (256/352) (*x*^2^ = 115.03; *p* < 0.0001; Cohen's *d* = 0.740). The pass rate for the CP certification examination for same-year candidates was 94.5% (3345/3538), whereas the pass rate of candidates who postponed the exam a year or more was 77.09% (175/227) (*x*^2^ = 106.80; *p* < 0.0001; Cohen's *d* = 0.904). The effect size for the AP certification examination showed a medium advantage toward same-year candidates, and the effect size for the CP certification examination showed a large advantage toward same-year candidates (Table 2).

The number of primary certification examinations candidates surveyed, number of responses, and response rate are presented in Table 3. There were too few respondents who chose to take the examination(s) in the Fall administration who responded to the survey to disclose themes. Responses from some of these individuals, however, did include a desire to have more time to study, as well as personal reasons for test postponement, such as life events (e.g., marriage). The theme from individuals who chose to take the certification examination(s) in the earlier administration largely reflected a desire to not have studying for the primary certification exam interfere with fellowship training, an expectation of their program to take the exam at that time, and the earlier administration being most convenient and coincident with the completion of training (Table 4).

## Discussion

The primary certification examinations that occur at the end of residency training are high-stakes events that evoke substantial stress.^1^ Pathology residents seeking certification from the American Board of Pathology have two opportunities per year to attempt primary certification. Although most pathology residents undertake their initial attempt at certification near the end of residency training (i.e., in the Spring of their graduating year), some individuals postponed the certification examination(s) until the Fall administration or later. This study demonstrated that, although psychometrically-equivalent examinations are given in the Spring and Fall administrations, individuals who delayed their initial attempt at pathology certification until the Fall administration were slightly more likely (i.e., a small effect size) to fail the examination(s) compared with individuals who took the earlier (i.e., Spring) administration. Similarly, individuals who postponed taking the primary certification exam(s) a year or more were significantly more likely to fail compared to those who took the primary certification examinations(s) in the year in which they graduated (i.e., a moderate effect size for AP and a large effect size for CP).

The association between a delay in testing and poorer outcomes has been documented in a variety of settings. Several researchers have demonstrated that the closer that particular medical school rotations were taken to the corresponding certifying examinations (e.g., NBME Part II), the higher the subtest scores for these rotations.^2^, ^3^, ^4^ An association between delayed testing and a higher likelihood of failing a board-certification or qualifying examination has also been described by the American Board of Surgery (ABS), the American Board of Emergency Medicine (ABEM), and the American Board of Physical Medicine and Rehabilitation (ABPMR).^5^, ^6^, ^7^

Malangoni and colleagues from the ABS studied individuals who took the ABS qualifying examination immediately after residency versus those who delayed taking the examination for a year or more. The pass rates decreased from 87% in the immediate cohort to 57% for individuals who delayed testing by 1 year, to 48% for delays of 2 years or more (p < 0.001).^5^ These results remained statistically significant even after controlling for in-training assessment scores. Researchers from the ABEM reported similar findings.^6^ They reported a pass rate of 98.9% for individuals who took the examination immediately after training, a 95.6% pass rate after one year delay (p = 0.003), and an 86.6% pass rate if the delay was 2 or more years (p < 0.001).^6^ These results were also controlled for in-training assessment scores. Finally, the ABPMR reported similar findings for their two-part certification examination.^7^ The pass rate of Part I of their examination was 91% immediately after training, which decreased to 68% after a one-year delay, then to 59% for 2 or more years of delay; the trend in the Part II exam was similar (i.e., 90% immediate, 83% with one year delay, and 68% with ≥2 year delay).

The reasons for these findings are likely multifactorial, but the fact that ABS and ABEM controlled for in-training assessment scores suggests that the differences are due to more than just academic ability. It was hypothesized that these changes, in part, may be simply due to a degradation of knowledge with respect to time away from relevant training (i.e., forgetting).^5^^,^^8^^,^^9^

A more complex relationship may exist between test anxiety, academic procrastination, and test performance. A significant relationship has been demonstrated between academic procrastination and test anxiety, which includes fear of humiliation (e.g., failure).^10^^,^^11^ Additionally, increased stress levels, particularly those associated with intrusive thoughts and feelings of impending failure, have been correlated with poor test performance.^12^ Another study has demonstrated that an increased stress response during a high-stakes exam, as measured by changes in cortisol levels, was associated with a test score that was 0.40 standard deviations lower than expected.^1^ It is, therefore, conceivable that individuals with a high level of stress may procrastinate (i.e., postpone their certification exam) and have a poorer pass rate from a combination of both an increased stress response, as well as from the degradation of knowledge over time.

The survey of individuals who took the primary certification examination(s) in 2021 and 2022, unfortunately, had too few responses from individuals who took the examination(s) in the Fall administration to draw any meaningful conclusion. However, some did report that they postponed the exam(s) to have more time to study, which could have been secondary to increased test anxiety or simply an introspective assessment of being less than adequately prepared. The survey did, however, disclose reasons that individuals took the examination(s) at the earlier administration. These reasons included a desire to not interfere with future training (i.e., fellowship) by studying for the primary certification examination(s), a feeling of readiness, convenience, and taking the administration that coincided closest with the end of residency training. Importantly, many also disclosed that it was an expectation of their training program that they should take the examination(s) at the earlier administration.

The findings of this study support those of others that individuals who take their certification examination at the administration nearest to the end of training are more likely to be successful compared with individuals who postpone the examination. Program directors should encourage their trainees, who they believe are ready, to take the examination at the administration nearest to the end of training to have the highest likelihood of success.

## Conflict of interest and funding statement

Internal funding was used for this project. All authors, except Dr. Swerdlow, are employees of the American Board of Pathology.
